# Specific Features of the Ukrainian Urban Adolescents' Physical Activity: A Cross-Sectional Study

**DOI:** 10.1155/2020/3404285

**Published:** 2020-04-09

**Authors:** Olena Yelizarova, Tetyana Stankevych, Alla Parats, Michael Antomonov, Nadiya Polka, Svitlana Hozak

**Affiliations:** State Institution “O. M. Marzieiev Institute for Public Health”, National Academy of Medical Sciences of Ukraine, Kyiv, Ukraine

## Abstract

**Background:**

An increase in the proportion of physically active peoples is one of the public health priorities. Particular attention should be addressed to the adolescent group with regard to their sensitivity. The purpose of our study is to determine the overall level of physical activity (PA) of urban adolescents and to evaluate its components.

**Methods:**

The study included the results of a survey of 415 children aged 11–15 years from public schools in Ukraine. We have adapted the standardized questionnaire QAPACE, which allows characterizing the level of adolescent's PA by indicators: type, duration, and intensity.

**Results:**

A sedentary lifestyle is typical for most Ukrainian urban adolescents. Only 30.8% of Ukrainian urban adolescents (45.4% of boys and 21.4% of girls; *p* < 0.001) meet the recommended level of moderate-to-vigorous PA (at least 60 minutes per day). The chances to follow the recommended moderate-to-vigorous PA are almost 5 times higher in the group of adolescents exercising at their own wish and not by parents' direction (OR = 4.96; 95% CI: 2.77–8.90; *p* < 0.001). Girls have a 3.0 higher chance of not adhering to recommendations for duration of PA (OR = 3.01; 95% CI: 1.95–4.63). They are 2.5 times more likely to lead a sedentary lifestyle (OR = 2.48; 95% CI: 1.54–3.98) than boys.

**Conclusion:**

The obtained results are indicative of a lack of implementation of measures to increase physical activity, which requires public attention to this problem. A search for ways to improve public policy towards optimizing adolescents' PA is still an urgent goal for scholars and practitioners. PA-promoting measures should be developed taking into account the impact of family traditions, accessibility of sports facilities, leisure, advertising, and other factors. The obtained results are the initial stage for developing the program of physical inactivity prevention.

## 1. Introduction

An increase in the proportion of physically active adolescents is one of the public health priorities because physical activity (PA) is associated with promoting lifelong health and prevention of risk factors for various health conditions, including cardiovascular, musculoskeletal, and mental disorders, obesity, and type 2 diabetes [1–3]. Numerous health benefits allow considering PA a powerful tool for the prevention of noninfectious chronic disease. On the contrary, physically inactive adolescents tend to have decreased cognition (e.g., academic performance and memory) [4], higher body fat [5], and worse health [6, 7] compared to their physically active same-age peers. Therefore, in order to identify the reasons for the modification of adolescent PA and to develop measures for the prevention of noninfectious chronic diseases, an objective study of this group, taking into account the peculiarities of lifestyle, is necessary [8].

In Ukraine, to increase the physical activity of adolescents and the formation of their motivation for a healthy lifestyle, programs such as “The State Targeted Social Program for the Development of Physical Culture and Sports for the period up to 2020” and “The National Strategy for the Improvement of Physical Activity in Ukraine for the period up to 2025–physical activity is a healthy lifestyle-a healthy nation” are directed [9]. According to reports of these programs' implementation, in 2018, only 46.7% of students in schools covered recreational and physical exercise activities.

Along with relatively low rates of physical activity among Ukrainian adolescents, according to the Ministry of Health of Ukraine, there is an increase in the prevalence of chronic diseases [10]. A thirty-year analysis of the disease prevalence in Ukraine conducted by Volosovets et al. shows an increase of 36.1% [11]. Our own scientific studies, related to studies of health of the children, show that the proportion of children with chronic diseases in children of primary school and high school students is 1.7 times different [12]. Taking into account the current negative health trends, a further search for ways to increase physical activity in Ukrainian population is relevant. Particular attention should be addressed to the adolescent group with regard to the environmental sensitivity. However, to our knowledge, no prior studies have focused on the physical activity of Ukrainian urban adolescents in terms of their lifestyle and motivational factors.

The purpose of our study is to determine the overall level of PA of urban adolescents and to evaluate its components.

## 2. Materials and Methods

### 2.1. Study Design

#### 2.1.1. Setting

This study was carried out within the framework of the project titled “Scientific substantiation of criteria for optimal physical activity of secondary school-age population”. The project began in 2017 after the approval of the Bioethics Committee of the SI “O.M. Marzieiev Institute for Public Health” (Protocol No. 1, May 25, 2016).

Criteria for inclusion in the study were as follows:

- Age 11–15 years
- No acute infectious disease within 3 months before the survey
- No physical traumas at the time of the study
- No chronic disease exacerbation within 3 months prior to the study
- Parental informed consent for the study
- The adolescents' understanding that the daily routine as of the moment of the survey was habitual for them
- All requested fields of the questionnaire completed

Given that the behavioral factors of megapolis and town residents could differ, which would result in bias, the survey was carried out in three different cities of Ukraine, including Kyiv, Sumy, and Pereiaslav-Khmelnitskyi.

State-owned schools utilizing typical educational curricula became the object of the study. According to the State Statistics Committee, 96.2% of Ukrainian city schoolchildren attend such schools. Schools were selected using a random number generator. Living standards in the areas where the students of the selected schools live corresponded to those 85.7% of the population. The areas did not differ by the criteria of environmental pollution. The data regarding living standards and environmental conditions were obtained from open sources of information (official state statistical online resources). Grades for the study were chosen by alternation “A,” “B,” “C,” “D,” and “E” in each school. For example, at school No. 258, grades were selected such as 5A, 6B, 7C, 8D, and 9E.

After obtaining permission from school principals to conduct the survey, there were held parent meetings where certified specialists and school authorities explained the purpose of the survey and invited the children to participate in it. At the end of the meeting, the parents received information booklets, the form of informed consent, and a questionnaire. Upon obtaining the parental informed consent, separate meetings were held for adolescents to explain the purpose of the study, analyze the questions included in the questionnaire, and define the concepts of “physical activity” and “physical exertion”. Consequently, our respondents gained additional knowledge about the importance of physical activity for health. Each questionnaire was assigned a code to enter data into the database. Children's surnames were not included in the database.

### 2.2. Data Sources/Measurement

To reach the goal, we have adapted the questionnaire Barbosa et al. QAPACE (Quantization de L'Active Physique en Altitude Chez le Enfants), which allows to characterize the level of adolescent's PA by indicators: type, multiplicity, duration, and intensity [13]. QAPACE provides high reliability (reproduction in repeated testing with correlation coefficients of 0.95–0.98) and validity (high level of correlation with an indicator of maximum oxygen consumption with correlation coefficients of 0.78–0.80) [13]. For use in our study, the QAPACE questionnaire was translated into Ukrainian, adapted to the national characteristics of the educational process and leisure of adolescents, and expanded with questions regarding the attitude of children to their physical activity, sports preferences, and interest in physical education and sport section. Supplementary questions were developed together with the psychologist. They had cross-answers to test for honesty. The vacation activities question pool was excluded from this questionnaire as we planned to address this area in further studies. The religious activities block was also excluded. Questionnaire categories are summarized in Table 1.

After the translation of the questionnaire, at the first stage of the QAPACE standardization for use in the Ukrainian population, we offered to answer the questions to a small group of adolescents aged 11–15 (*n* = 10) in order to clarify the question wording and the relevance of some items inclusion. This survey gave us the opportunity to improve the wording in the Ukrainian language and make it more understandable. For example, in the section “out-of-school activities,” there were included visits to large shopping malls with the selection of leisure activities. A large number of urban adolescents spend a lot of time there, being involved in both moderate physical activities, which take them a considerable number of minutes (bowling, videos, and active games on dance simulators), and in activities which take sedentary activity (cafes, cinema, talking with friends, Internet surfing, and gadget games).

At the second stage of the QAPACE standardization, 66 students aged 11–15 were asked to fill out the questionnaires twice with an interval of one week. This test-retest measures allowed us to obtain high intraclass correlation coefficient (ICC) (0.88–0.98) within each category and confirm the reliability of the QAPACE questionnaire (Table 1).

Internal consistency of answers to questions enabling to understand the motivation of adolescents regarding MVPA activities was found by calculating Cronbach's alpha. The value of Cronbach's alpha in the initial sample (*n* = 66) was 0.87. In the final sample (*n* = 415), Cronbach's alpha was 0.77, which was acceptable for including these answers and discussing them. When evaluating the reliability of motivational responses consistency with the real level of MVPA, Cronbach's alpha was 0.72.

The results for the weekly duration of all types of activity and sleep were summarized up to 24 hours using the arithmetic mean.

The structure of the 24 hours was determined by the type of energy expenditure and duration of each activity and sleep. Sedentary behavior (SB) was attributed to daytime activity with a sitting or reclining body position and energy expenditure ≤1.5 metabolic equivalents (MET) [14]. According to the Global Recommendations on Physical Activity for Health, WHO light physical activity (LPA) was higher than the basal metabolic rate by 1.51–2.9 times, moderate physical activity (MPA) by 3.0–6.9 times, and vigorous physical activity (VPA) 7.0 times or more [8]. In addition to certain types of activity, a total level of moderate-to-vigorous physical activity (MVPA) was calculated.

The survey obtained demographic characteristics (gender and age) and anthropometric characteristics (weight and height). Body mass index was calculated by the WHO method.

The physical activity level (PAL) and total energy expenditure (TEEdaily) were calculated based on the obtained demographic and anthropometric characteristics, data on the duration, and multiplicity of elements of the behavioral factors.

The assessment of lifestyle intensity from the point of view of physical activity was carried out according to WHO recommendations [15]. In point terms, PAL values for the age group of 10–15 years are for the sedentary lifestyle 1.41–1.71, moderate lifestyle 1.72–2.01 points, and vigorous lifestyle 2.02–2.42 points.

To calculate daily energy expenditure in adolescents, we used the Compendium of Energy Expenditures for Youth [16].

When calculating the TEEdaily, the MET values of various activities from the compendium were multiplied by the resting metabolic rate (RMR) estimated based on Schofield's age-, gender-, and mass-specific prediction equations, by body weight in kg and by the number of minutes activity performed: TEEdaily = MET value × RMR (kcal·kg^−1^·min^−1^) × kg body weight × number of minutes activity performed.

The technique is described in detail and recommended for use in [16].

To compare actual total energy expenditure (TEEdaily) with “ideal” (TEE) was modeled according to Torun's method [15] adjusting by body weight and gender.

### 2.3. Study Size

As of January 1, 2017, there were 1,252,220 people aged between 12 and 15 in Ukraine. A representative sample for the study of behavioral factors at *α* = 0.05 was 384 individuals. However, considering the likely errors in completing the questionnaire or refusal to be involved in the study, we invited 550 people to participate in the study. In the beginning, 507 parents gave the informed consent, but in the course of the study, 60 of them refused to have the children surveyed. Consequently, only 447 questionnaires were received. Four hundred and fifteen of them (163 boys and 252 girls) met the eligibility criteria. Anthropometric characteristics were taken from 385 participants (149 boys, 236 girls) who were enrolled in the study. Four hundred and eleven respondents provided the information on parental education and family income.

### 2.4. Statistical Methods

Descriptive statistics elements (sample means and standard deviation) were used to describe the duration of sleep, SB, LPA, MPA, and VPA. Welch's *t*-test was employed to study unequal variances. Classic Student's *t*-test was used for unequal variances. All obtained values had normal distributions according to the Kolmogorov–Smirnov test (*p* > 0.1). To test the *H*_0_ hypotheses of values across age groups, we used a one-way ANOVA separately for boys and girls. Cross-tabulations were built to study the distribution of PAL selection. Differences between groups were determined using Pearson's chi square. The significance of the impact of parental education level, family income, and gender on the recommended levels of physical activity was determined using the log-linear analysis for frequency tables and confirmed by chi square. The model was considered relevant when confirming the *H*_0_ hypothesis (*p* > 0.05).

Statistical processing was performed using STATISTICA 8.0. The significance level was set at 0.05.

Odds ratio (OR) was calculated using the Odds ratio calculator programme of MedCalc Version 19.0.7.

## 3. Results

### 3.1. Participants

General characteristics of the sample are presented in Table 2. The study sample consisted of 415 Ukrainian adolescents aged 11–15 years, including 83 individuals aged 11 years (34 boys and 49 girls), 114 students aged 12 years (50 boys and 64 girls), 83 students aged 13 years (31 boys and 52 girls), 69 students aged 14 years (28 boys and 41 girls), and 66 students aged 15 years (20 boys and 46 girls).

The characteristics of the physical development of the study participants by gender and age group are presented in Table 3. The obtained results correspond to the expected gender-age trends of adolescent development.

No statistically significant difference was found between the groups of boys and girls by parental education level, family income, and place of residence (*p* > 0.2).

### 3.2. Main Results

**Figure 1:**
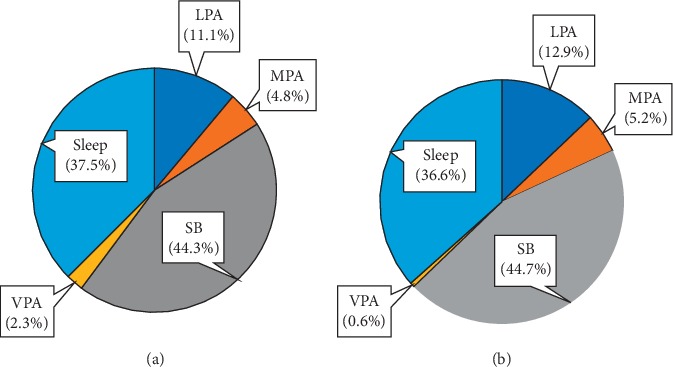
Structure of a typical day (24 hours) of a secondary school-age student (%): (a) boys; (b) girls.

The study showed that, during the 24 hours of urban adolescents, sleep takes 36.6–37.5% of the time, SB–44.3–44.7%, LPA–11.1–12.9%, MPA–4.8–5.2%, and VPA–0.6–2.3% (Figure 1).

Table 4 provides information on the duration of different types of daily activity. In boys, SB continues 640.7 ± 5.9 min/day and in girls 645.4 ± 5.5 min/day (*t* = 0.6; *p*=0.57). The duration of LPA is higher for girls than for boys by 14.1% (*t* = 4.5; *p* < 0.001), while the VPA is higher four times for boys than for girls (*t* = 8.4; *p* < 0.001). The daily number of minutes for MVPA is higher for boys than for girls by 22.2% (*t* = 5.9; *p* < 0.001).

Log-linear analysis was performed to test the effect of variables such as “parental education level,” “income,” and “gender” on compliance with the recommended MVPA time (Groups_MVPA) as defined in the WHO guideline. Groups_MVPA included two grades: 1 = less than 60 min/day of MVPA and 2 = more than 60 min/day of MVPA. The parental education level was subdivided into three grades: 1 = secondary education, 2 = secondary vocational education, and 3 = higher education. The income had three grades: 1 = below average, 2 = average, and 3 = above average. The null hypothesis was true for factors such as “Gender” and “Groups_MVPA” (Table 5).

When assessing the effect of variables, both separately and in combination, 14 models were calculated using log-linear analysis, but only one combination was found significant: gender/groups MVPA (*χ*2 = 21.4; *p* < 0.001). Using *χ*2 values, it was revealed that the impact of gender on the compliance with MVPA recommendations was 74.9%, and 25.1% was explained by the influence of other factors (Table 6).

More than 60 minutes of moderate and high physical activity have 30.8% of respondents in the general study group (45.4% boys and 21.4% girls; *p* < 0.001). Boys are more likely to meet the recommended physical activity than girls (OR = 3.01; 95% CI: 1.95–4.63; *p* < 0.001) (Table 6).

It is determined that the chances following the recommended MVPA are almost 5 times higher in the group of children who exercise at their own wish and not by parents' direction (OR = 4.96; 95% CI: 2.77–8.90; *p* < 0.001) (Table 7).

Sedentary lifestyle is typical for 72.5–80.4% of urban children of secondary school-age, moderate lifestyle 13.7–23.2%, and vigorous lifestyle 1.4–5.9% (Table 8). PAL gradations are statistically different by sex (*χ*^2^ = 16.2; *p* < 0.001). In the boys' group (*n* = 149), sedentary lifestyle leads by 65.1 ± 3.9% of children, moderate lifestyle by 28.2 ± 2.4%, and vigorous lifestyle by 6.7 ± 2.0%. The corresponding values in the girls' group (*n* = 236) are 82.2 ± 2.5%, 16.1 ± 2.7%, and 1.7 ± 3.9%.

It is determined that girls' chances of sedentary lifestyle were 2.5 times higher (OR = 2.48; 95% CI: 1.54–3.98; *p* < 0.001) than boys (Table 7). Therefore, during the developing measures to increase adolescent PA, it is necessary to consider this feature.

It is established that the urban adolescents' energy expenditures per day is from 1475.8 to 4055.1 kcal per day with an average value 2215.9 ± 22.1 kcal/day. Boys expenditures are 17.9% more kcal than girls (*t* = 11.4; *p* < 0.001).  Table 9 presents the modeled (TEE) and actual (TEE_daily_) average daily energy expenditures. An analysis of the data showed that actual daily energy expenditures are 9.8% lower than those modeled in the boys and for 13.1% are lower in the girls (*p* < 0.001).

## 4. Discussion

Despite the limited number of participants in the study, we managed to identify major trends in the behavioral factors of Ukrainian urban adolescents. Probable biases of the research results due to inaccuracies in completing the questionnaire were eliminated through additional interviews. A limitation of the study is also the established framework for the study of physical activity only across the training period, including days off, but excluding the holiday period. The vacation period will be further addressed in publications focusing on the significant decrease in adolescent physical activity while away from school [17, 18].

A study showed that SB was dominant in the structure of daytime activity of the urban adolescents. Comparison of the daily activity pattern of children with similar studies by Finnish and Japanese scientists showed that the SB duration of Ukrainian adolescents is the highest [19, 20], while the VPA level is 2.0–3.6% in all three countries. The level of LPA of Ukrainian adolescents is the same as that of Finland. The average minutes spent by adolescents in Finland for SB is 513 min/day, LPA 144 min/day, MPA 131 min/day, and VPA 20 min/day [20]. In Japan, the study of PA in children and adolescents revealed 441.4 min/day for SB, 307.1 min/day for LPA, 34.6 min/day for MPA, and 28.3 min/day for VPA with total daily minutes activity of PA 811.2 min/day. This study reports that most children of primary school-age are given adequate physical activity, while among adolescents, the recommended level of PA does not reach about 70% of children [19]. China's study found that teens in China spend an average of 521.50 ± 110.02 minutes per day on SB [21]. Verloigne et al. report an SB duration of 268 min to 506 min in European adolescents [22]. Researchers in other countries indicate SB duration ranges from 219.8 to 521.5 min/day, but in our country, this rate is highest.

According to WHO guidelines for reducing the risk of developing noncommunicable diseases, moderate-to-vigorous physical activity should be at least 60 minutes per day [8]. However, scientific observations of physical activity of adolescents from 105 countries suggest that about 80.0% of children do not reach the recommended level [23]. Daily MVPA minutes for Ukrainian adolescents are lower than WHO recommended, but higher than the 33.3 min/28.3 min recorded in 12–15-year-old Americans and Chinese [24]. According to another study in the US, “Growing Up Today Study,” the average MVPA was 22.0 min per day. Girls have a 57.0% lower chance of meeting the recommended MVPA minutes than boys [25]. The proportion of children in Boston (USA) who adhere to the recommended 60 min/day is 34.0% [26].

However, according to several studies, there are groups in which an average MVPA of more than 100 min/day has been detected. Thus, a study of Mexican adolescents found that the weekly duration of MVPA in boys is 110.6 min/day and in girls is 105.5 min/25 [27]. In New Zealand, during the program “The Built Environment in Adolescent New Zealanders,” it is found that adolescents had MVPA of 114 min/day, with an SB value of 354 min/day with total minutes of activity (828 min/d) [28].

The statistics of Healthy Lifestyle in Europe by Nutrition in Adolescence (8 European Countries: France, Belgium, Hungary, Spain, Germany, Greece, and Austria) indicate that MVPA more 60 minutes per day level have 30.7–34.1% of adolescents [29]. A research conducted in Finland shows that 31.7% of adolescents (37.9% in boys and 25.5% in girls) had MVPA more 60 minutes per day [20]. In Canada, 39.0% of adolescents (45.0% boys and 30.0% girls) meet the recommendations [30]. It corresponds to Ukrainian levels.

Some researchers report that the level of parental education and family income are factors positively associated with the level of motor activity and adolescent health. They contribute to the gain of the recommended 60 min/day MVPA [31–33]. However, other studies demonstrate a lack of relationship between these variables or show the presence of the relationship with leisure time PA [34–36]. In our study, there was no statistically significant difference between the groups of male and female adolescents regarding the parental education level and family income and gaining the recommended 60 min/day MVPA. Additional interviews with parents showed that their awareness of the need for physical activity for a growing body did not depend on the level of their education or family income. That is, health education of the Ukrainian population of is one of the problems requiring solution at the state level.

A comparison of the modeled and the actual daily energy expenditures showed that expenditures of secondary school-age children are 9.8–13.1% kcal less than that recommended, which in the future may lead to overweight. The obtained statistics are comparable to the results obtained by Ishchenko in the study of physical activity of urban adolescents in 2012 [37]. But it is noteworthy that, with a steady increase in physical development (Table 2), in the group of girls, TEEdaily hardly changes from 11 to 13 years and increases only from 14 to 15 years. Boys had a higher daily energy expenditure than girls (*t* = 10.1; *p* < 0.001), which is consistent with data from other researchers who used the QAPACE questionnaire [38, 39].

Definitely, sedentary lifestyle and insufficient daily energy expenditures are common to Ukrainian urban adolescents. During the developing measures to increase adolescent PA, it is necessary to consider girls' higher chances of sedentary lifestyle and lifestyle risk behaviors [23]. For example, according to Baharudin et al., girls' chances of sedentary lifestyle are 2.9 times higher than boys (OR = 2.9; 95% CI = 2.66–3.10) [40]. A study by Teh et al. show that adolescent girls are also more likely to be lifestyle risk behaviors than boys (OR 2.82; 95% CI: 2.32–3.43) [41]. According to data retrieved using the QAPACE questionnaire by Barbosa et al., urban teenage girls are less active than boys [38].

We believe that selection bias has been avoided owing to the random selection of experiment participants from typical Ukrainian educational institutions. After all, the purpose of this study was to characterize the overall trends in the population of Ukrainian adolescents aged 11–15 years. What is more, the validity of the results was not impeded by the disproportionate composition of the gender sample, as the results were described separately for boys and girls. Given the respondent sample, survey tools, and statistical analysis, the results obtained are valid and the findings can be extrapolated to the entire population of this age group in the country. However, for better generalisability and the development of measures to prevent physical inactivity, a larger scale study involving other regions of the country is needed.

The obtained results are indicative of a lack of implementation of measures to increase physical activity, which requires public attention to this problem. A search for ways to improve public policy towards optimizing adolescents' PA is still an urgent goal for scholars and practitioners. PA-promoting measures should be developed taking into account the impact of family traditions, accessibility of sports facilities, leisure, advertising, and other factors. As adolescents spend a significant portion of their lives at school, one of the main objectives in improving their PA is to optimize physical education and physical activity in school and involve a larger proportion of adolescents in school sports sections.

## 5. Conclusion

A sedentary lifestyle is typical for most Ukrainian urban adolescents. Only 30.8% of Ukrainian urban adolescents (45.4% of boys and 21.4% of girls; *p* < 0.001) meet the recommended level of MVPA as specified in the WHO guidelines (at least 60 minutes per day). The chances to follow the recommended MVPA are almost 5 times higher in the group of adolescents exercising at their own wish and not by parents' direction (OR = 4.96; 95% CI: 2.77–8.90; *p* < 0.001).

Among Ukrainian adolescents, time spent sedentary is one of the highest in the world (640.7 ± 5.9 min/day for boys and 645.4 ± 5.5 min/day for girls). Girls have a 3.0-fold higher chance of not adhering to recommendations for duration of daily physical activity (OR = 3.01; 95% CI: 1.95–4.63). They are 2.5 times more likely to lead a sedentary lifestyle (OR = 2.48; 95% CI: 1.54–3.98) than boys.

We revealed that the levels of adolescents' energy expenditures were by 9.8–13.1% kcal lower than those defined in the WHO guidelines, given the specific features of physical development.

The obtained results are the initial stage for developing the program of physical inactivity prevention. At the next stage, we are planning to determine the level of optimal physical activity (dose-effect) for Ukrainian urban adolescents, taking into account the medical and social aspects.

## Figures and Tables

**Table 1:** Categories of physical activity in our customized QAPACE questionnaire.

Category	Questions	Explanation	MET	ICC^*∗*^
1	1, 2	Sleeping	0.9	0.93
2	3	Toilet	2.5	0.93
3	4–6	Meals	1.5–2.0	0.96
4	7, 8	Transportation to school	1.4–6.2	0.98
5	9, 10	Classroom (class hours and homework)	1.4	0.94
6	11–13	Mandatory physical education	4.2	0.88
7	14–22	Other activities in school (sports, artistic activities, and art)	1.6–10.0	0.98
8	23–24	Transportation to out-of-school activities	2.9	0.97
9	29–36	Out-of-school activities in school time (watching TV, listening music, reading, listening music, sports, and art)	1.2–10.0	0.98
10	37–44	Out-of-school activities in weekend (watching TV, listening music, reading, listening music, sports, art)	1.2–10.0	0.95
11	45–50	Domestic activities in home	1.8–4.2	0.97
12	51–59	Supplementary questions:	—	0.91
		Do you think you have enough physical activity?		
		If “not enough,” what prevents you from being physically active?		
		Was your physical activity typical over the last week?		
		If it was “lower than usual,” what prevented you?		
		Do you class or your friends believe it is trendy to do sports?		
		What kind of sport do you think is trendy?		
		Imagine that you can play any sport. Which will you choose?		
		Is there anything that prevents you from playing your dream sport? If yes, specify what exactly.		

*Note.* ^*∗*^ICC, quantifying test-retest reliability the QAPACE questionnaire using the intraclass correlation coefficient.

**Table 2:** Characteristics of the participants.

Variable	Boys		Girls		*t* value	*p*
	Means	SD	Means	SD		
Age, years (*n* = 415)	12.7	1.3	12.9	1.4	−1.4	0.150
Weight, kg (*n* = 385)	47.1	12.1	46.1	10.3	0.8	0.409
Height, m (*n* = 385)	1.60	1.24	1.59	1.0	1.1	0.281
BMI, kg/m^2^ (*n* = 385)	18.3	2.9	18.2	2.7	0.4	0.664

*Note.* Means, sample means; SD, standard deviation; *t* value, Student's *t*-test; BMI, body mass index.

**Table 3:** Characteristics of physical development of research participants by gender and age group.

Age (years)	Weight (kg)			Height (cm)		BMI (kg/m^2^)	
	*N*	Means	SD	Means	SD	Means	SD
Boys	149	47.1	12.0	160.2	12.4	18.3	2.8
11	30	40.0	9.4	151.3	8.5	17.8	2.7
12	44	41.5	9.2	153.9	8.5	17.7	2.8
13	31	48.1	8.8	162.2	10.3	18.3	3.2
14	26	52.6	10.3	165.1	9.8	19.0	2.6
15	18	62.0	11.1	176.6	9.0	19.8	2.5
*F*		24.10		31.87		2.80	
*p*		<0.001		<0.001		0.028	

Girls	236	46.3	10.1	158.8	9.4	18.3	2.7
11	47	39.0	8.0	149.0	7.3	17.5	2.6
12	59	41.4	7.9	154.4	7.6	17.4	2.4
13	47	47.4	10.2	161.4	8.4	18.2	3.3
14	40	51.8	7.8	163.9	4.8	19.2	2.6
15	43	53.7	7.9	166.4	5.8	19.3	2.1
*F*		28.52		51.26		6.40	
*p*		<0.001		<0.001		<0.001	

*Note. F*, Fisher–Snedecor distribution for *H*_0_ checking between age groups; SD, standard deviation; BMI, body mass index.

**Table 4:** Duration of adolescents' daily activites.

Activity	Gender	Means	*n*	SD	*t*	*p*
SB (minutes/day)	Boys	640.7	163	75.8	0.6	0.57
	Girls	645.7	252	86.8		

LPA (minutes/day)	Boys	159.9	163	56.4	4.5	<0.001
	Girls	186.1	252	59.4		

MPA (minutes/day)	Boys	69.0	163	46.5	1.2	0.25
	Girls	74.5	252	47.5		

VPA	Boys	33.9	163	39.9	8.4	<0.001
	Girls	8.1	252	22.1		

MVPA^*∗*^ (minutes/day)	Boys	63.1	163	44.7	5.9	<0.001
	Girls	49.1	252	34.9		

*Note.* ^*∗*^More than 60 min/day MVPA achieves 45.4% of boys (*n* = 73) and 21.4% of girls (*n* = 54) (*p* < 0.001); SD, standard deviation.

**Table 5:** Results of fitting all K-factor interactions.

K-factor	Degrees of freedom	Maximum likelihood (chi sq.)	Probability	Pearson (chi sq.)	Probability
1	7	737.1	0.001	1327.9	0.001
2	17	43.9	0.001	51.1	0.001
**3**	**17**	**10.8**	**0.865**	**10.9**	**0.859**
**4**	**6**	**3.2**	**0.786**	**3.4**	**0.757**

Factor codes: 1 = level of education of parents; 2 = income; 3 = gender; 4 = Groups_MVPA.

**Table 6:** Tests of marginal and partial association.

Effect	Degrees of freedom	Partial association chi square	Partial association *p*	Marginal association chi square	Marginal association *p*
1	3	454.1	0.000	454.1	0.001
2	2	198.5	0.000	198.5	0.001
3	1	20.6	0.000	20.6	0.001
**4**	**1**	**63.8**	**0.000**	**63.8**	**0.001**
12	6	15.5	0.017	15.1	0.019
13	3	0.7	0.877	0.8	0.857
14	3	2.9	0.405	2.5	0.474
23	2	4.1	0.130	3.5	0.170
24	2	1.3	0.511	0.3	0.852
**34**	**1**	**21.4**	**0.000**	**20.9**	**0.001**
123	6	2.8	0.830	2.9	0.820
124	6	3.8	0.708	3.6	0.733
134	3	1.7	0.642	2.3	0.521
234	2	2.4	0.308	2.4	0.305

Factor codes: 1 = level of education of parents; 2 = income; 3 = gender; 4 = Groups_MVPA.

**Table 7:** Odds ratio for adolescents meeting the recommended levels of MVPA (more than 60 min/day).

	%	OR	95% CI
*Gender*			
Boys	45.4	3.01	1.95–4.63
Girls	21.4		

*Motivation for MVPA*			
Own wish	48.1	4.96	2.77–8.9
Parents' direction	15.7		

*Note.* Odds ratio calculated for boys compared to girls and for group “own wish” compared with group “parent's direction.”

**Table 8:** Odds ratio for physical activity levels.

PAL	Boys (%)	Girls (%)	OR	95% CI
Sedentary lifestyle	65.1	82.2	2.48	1.54–3.98
Moderate lifestyle	28.2	16.1	0.49	0.30–0.81
Vigorous lifestyle	6.7	1.7	0.47	0.15–1.52

**Table 9:** Modeled and actual average daily energy expenditures (adjusting by weight and gender).

Age	*n*	TEE_daily_ (kcal)		TEE (kcal)		*t*	*p*
		Means	SD	Means	SD		
Boys	149	2488.8	488.8	2760.5	475.1	−4.9	<0.001
11	30	2416.4	479.2	2499.4	380.7	−0.7	0.461
12	44	2396.7	505.9	2548.6	396.2	−1.6	0.121
13	31	2404.5	467.4	2812.4	341.4	−3.9	<0.001
14	26	2616.1	435.4	2989.8	399.5	−3.2	0.002
15	18	2795.9	455.9	3293.0	508.4	−3.1	0.004
*F*		3.18		16.7			
*p*		0.015		<0.001			

Girls	236	2043.6	280.2	2352.7	237.5	−12.9	<0.001
11	47	2012.4	294.2	2157.1	227.2	−2.7	0.009
12	59	2013.0	290.8	2241.1	202.8	−4.9	<0.001
13	47	2012.7	289.1	2386.8	201.2	−7.3	<0.001
14	40	2120.6	224.8	2500.2	143.3	−9.0	<0.001
15	43	2081.5	280.7	2544.9	136.4	−9.7	<0.001
*F*		1.43		35.53			
*p*		0.226		<0.001			

*Note. F*, Fisher–Snedecor distribution for checking *H*_0_ between age groups; *t,* for checking *H*_0_ between TEE/TEEdaily rates; SD, standard deviation.

## Data Availability

The full census data used to support the findings of this study are restricted by the Bioethics Committee of the SI “O.M. Marzieiev Institute for Public Health” in order to protect privacy. The processed data are available from the corresponding author Olena Yelizarova upon request.
